# Comparative study of serum zinc concentration in recurrent herpes labialis patients and healthy individuals

**DOI:** 10.1186/s12903-020-01277-2

**Published:** 2020-10-28

**Authors:** Zahra Ranjbar, Maryam Zahed, Mohammad Ali Ranjbar, Zahra Shirmardan

**Affiliations:** 1 Oral and Dental Disease Research Center, Department of Oral and Maxillofacial Medicine, School of Dentistry, Shiraz University of Medical Sciences, Shiraz, Iran; 2 Oral and Dental Disease Research Center, Department of Oral and Maxillofacial Pathology, School of Dentistry, Shiraz University of Medical Sciences, Shiraz, Iran; 3 Student Research Committee, School of Dentistry, Shiraz University of Medical Sciences, Shiraz, Iran

**Keywords:** Herpes labialis, Zinc, Serum

## Abstract

**Background:**

Recurrent herpes labialis (RHL) is a common recurrent infective vesiculoulcerative disease. Topical and systemic administration of Zinc compounds has been indicated to have preventive and therapeutic effects. The purpose of this study was to evaluate the serum level of zinc in the patients with RHL and healthy individuals and also to investigate the correlation of this level with various parameters of the patient and disease course.

**Methods:**

This cross-sectional study was performed on 43 patients with a history of recurrent herpers labialis and 42 subjects without any previous experience of the lesion. Blood samples were taken, and serum zinc level was measured using colorimetry (spectrophotometry) method. Chi-Square test was used to compare the qualitative relationships, and for comparing the quantitative relationships, independent T-test was used. To observe the relationship of quantitative factors including serum zinc level, the number of relapses, and recovery rates, correlation test was taken.

**Results:**

The results show that, serum zinc level has no significant difference between healthy subjects and the patients (*p* > 0.05). Also, zinc level was not related to age and sex factors and frequency of relapse (*p* > 0.05). However surprisingly, there was a significant relationship between zinc level and recovery period in the RHL patients. The lower the serum zinc level, the higher the duration of recovery (*p* = 0.009).

**Conclusion:**

The results of this study indicate that, zinc deficiency can be considered as a risk factor for increasing the duration of herpes labialis lesions. Therefore, the evaluation of serum zinc level in the subjects with RHL and subsequent administration of zinc are recommended in these kind of patients.

## Background

Herpes simplex virus type 1 (HSV-1), as a member of the herpes virus family, is the etiologic factor of a variety of diseases such as primary gingivostomatitis, recurrent herpes labialis, and recurrent intraoral herpetic lesions. Accordingly, it generally affects the upper parts of the human body, and the primary infection appears after fluid or skin contact with contaminated individuals [1, 2]. In most of the cases, the virus tends to remain in the sensory ganglions or skin epithelium after the initial infection [3]. Factors such as sunlight, shock, stress, fever, and hormonal changes can trigger the reactivation of the virus causing a secondary or recurrent lesion like herpes labialis [1, 2].

Herpes labialis primarily appears as an itchy erythema and is followed within few minutes or hours by a group of small vesicles with clear fluid in the skin area around the oral cavity, lips, nose or the oral mucosa. The vesicles erupt and turn into multiple small superficial ulcerations that eventually merge forming a painful ulcer covered by a crust in following days. This lesion can be very irritating and disabling due to its pain and unappealing appearance [4, 5].

Drugs such as acyclovir and valacyclovir and also less common medications such as cytarabine and vidarabine, are used in the treatment of viral herpes infections [6, 7]. Acyclovir is widely used as a systemic agent or with topical application due to the severity of infection, and it is considered as the chosen treatment for herpes labialis infections [6]. Nevertheless, nausea, dizziness, headaches, mental changes, neurotoxicity and kidney disorders are the well-known side effects of this drug [8]. Nowadays, new treatments such as micro–nano filopodia-like Zinc oxide (ZnO) structures, cinnamon extract or prepared 3-O-sulfonated sugars are introduced to treat herpes simplex infections with fewer side effects [9–11].

Zinc is an essential feature for the natural function of cells and organs of the body [12–14]. The local use of zinc promotes the production of epithelial cells and endothelium of the vessels. In addition, the use of zinc compounds is found to enhance the local defense system [15]

Zinc has the capacity to stimulate cellular immunity. T-lymphocytes involved in cellular immunity are considered to be important in protecting against viral, fungal, and protozoic infections, as well as malignant and autoimmune diseases [16, 17]. Zinc appears to increase the number of the effecter and helper T-cells. On the other hand, Zinc deficiency has been shown to reduce host immunity by reducing circulating T-cells and decreasing the phagocytic activity of macrophages, which can generally affect cell-mediated immunity [18, 19].

The topical use of zinc solutions for the treatment and prevention of herpes simplex infections has long been established [17, 20, 21]. Furthermore, saliva zinc deficiency has been investigated as well as its role in the recurrence of this viral infection [22]. It has been shown that, salivary zinc is significantly lower among the patients suffering from the recurrent herpes labialis compared to healthy individuals [22].

Due to the fact that, serum zinc concentration is currently known as the best and most widely used biomarker of zinc status in populations [23], we decided to check this marker in the herpes labialis patients to further evaluate the effect of this element in the prevention and treatment of this recurrent lesion.

## Methods

This cross-sectional study was performed on 85 subjects including 43 patients with the history of recurrent herpes labialis (RHL) and 42 healthy individuals without any history of the disease, who were referred to the Oral and Maxillofacial Medicine department of Shiraz dental school in 2018. The sample size was chosen according to previous studies [22]. The study was approved by the ethical committee of Shiraz University of Medical Sciences. (IR.SUMS.REC.1397.449).

All subjects were over the age of 18 years old. The cases with the history of recurrent lesions were asked to visit an oral and maxillofacial medicine specialist in less than 48 h after the onset of a new lesion. Therefore, all patients were precisely diagnosed as having recurrent herpetic lesions. All the subjects were evaluated by a single examiner. A control group consisted of healthy subjects who were without any history of similar oro-facial and genital lesions during their life. This group was randomly selected from the subjects who attended the same clinic for routine dental checkup. They were matched in age and gender compared to the case group. Exclusion criteria for both groups were pregnancy, a history of systemic and immunological diseases affecting the activity of the immune system, and the use of drugs or any supplements. Furthermore, the subjects who experienced less than 3 recurrences in the past year or who had no clear memory of the number of relapses or exact duration of their last lesion were excluded from the patient group.

At the beginning of the study, an informed consent form was obtained from all the subjects. A form containing biographical information including age, gender, history of any certain disease, medications and supplements, the rate of relapse of the lesions in the past year, and the duration of recovery period of the last experienced lesion was obtained. Lesion improvement was considered when patient had no detectable lesion and no pain or other sensory changes like tingling existed. All blood samples were taken in the morning whilst fasting. The used method for measuring zinc levels was colorimetry (spectrophotometry). In this method, zinc forms a red chelate complex with 2-(5-Brom-2_pyridylazo)-5-(N-propyl-N-sulfopropylamino).

The results were statistically analyzed using SPSS software version 18. Chi-Square test was used to compare the qualitative factors. To compare the quantitative relationships, independent T-test was used. In addition, a Correlation test was taken, to observe the relationship between quantitative factors as follows: serum zinc level, number of relapses, and lesion improvement. To evaluate the sufficiency of sample size, power analyses were done after analyzing data. For this purpose, effect sizes (ES) were calculated and where the ES was medium to large, power was computed.

## Results

Of the participants in the study, 67 were women (78%) and 18 were men (22%). The mean age of the individuals with herpes lesions and the healthy subjects were 37.28 and 36.17 years old, respectively. There was no statistically significant difference between these two groups in terms of gender and age. (Tables 1 and 2).

**Table 1:** Number and percentage of gender difference in herpes and control groups

Groups	Gender		*p* value* (Student T-test)
	Men (number percentage)	Women (number percentage)	
Herpes	10 23.3%	33 76.7%	
Control	8 19%	34 81%	0.635

*p value < 0.05 is considered as significant

**Table 2:** The mean age of groups

Groups	Mean age (years old)	SD	Sig.(2-tailed)*
Herpes	37.28	9.192	0.636
Control	36.17	12.237	

*p value < 0.05 is considered as significant

To compare serum zinc levels, independent t-test was used. As a result, there was no significant difference in serum zinc level in the case and control groups (*p* = 0. 313) (Table 3).

**Table 3:** Average of Serum zinc level in herpes and control groups

Groups	Serum zinc average	SD	Sig.(2-tailed)*
Herpes	74.74	14.017	0.313
Control	71.86	12.139	

*p value < 0.05 is considered as significant

**Fig. 1:**
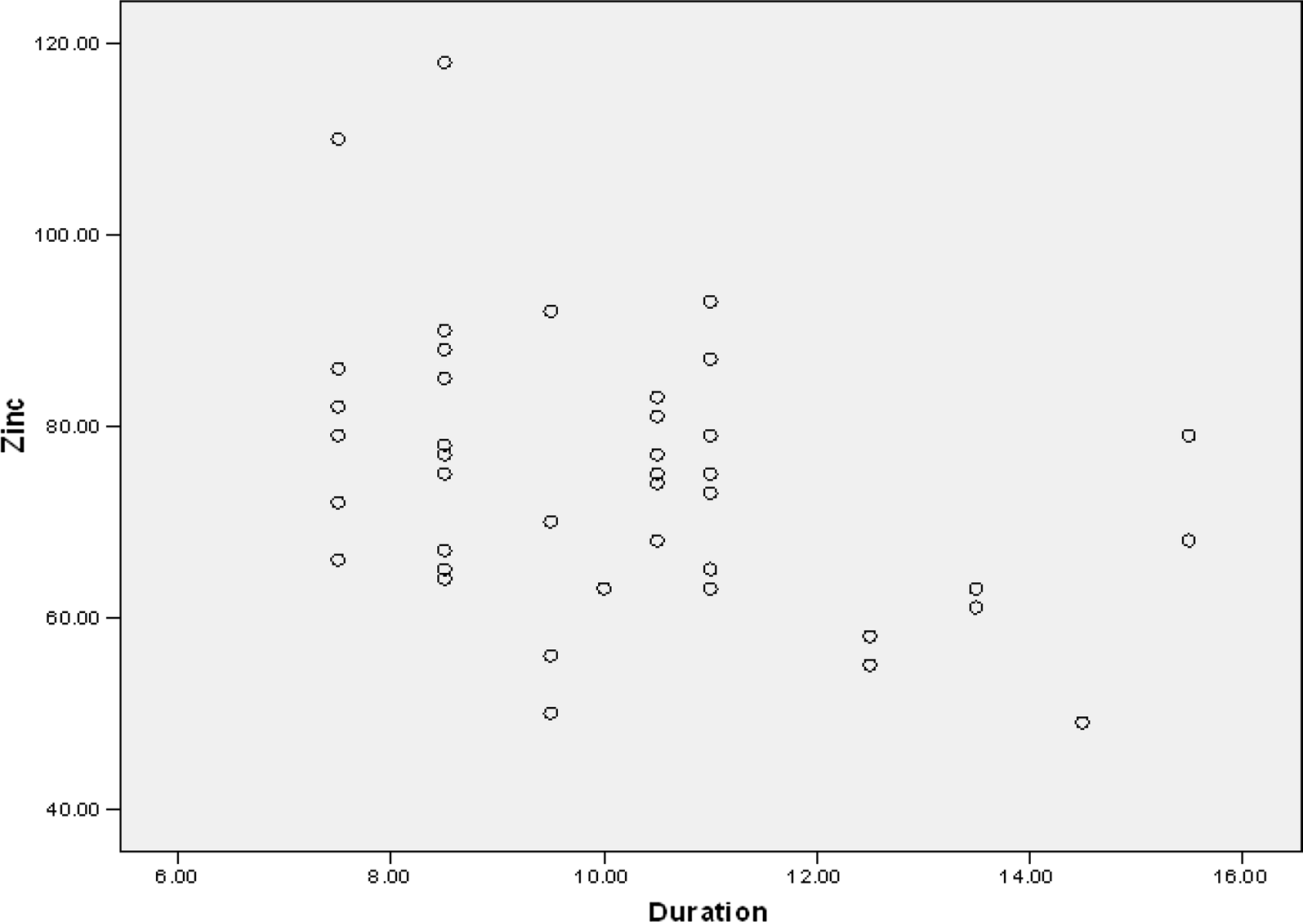
Correlation of serum zinc level (μg/dL) in comparison with the duration of lesion (days) in herpes cases

**Fig. 2:**
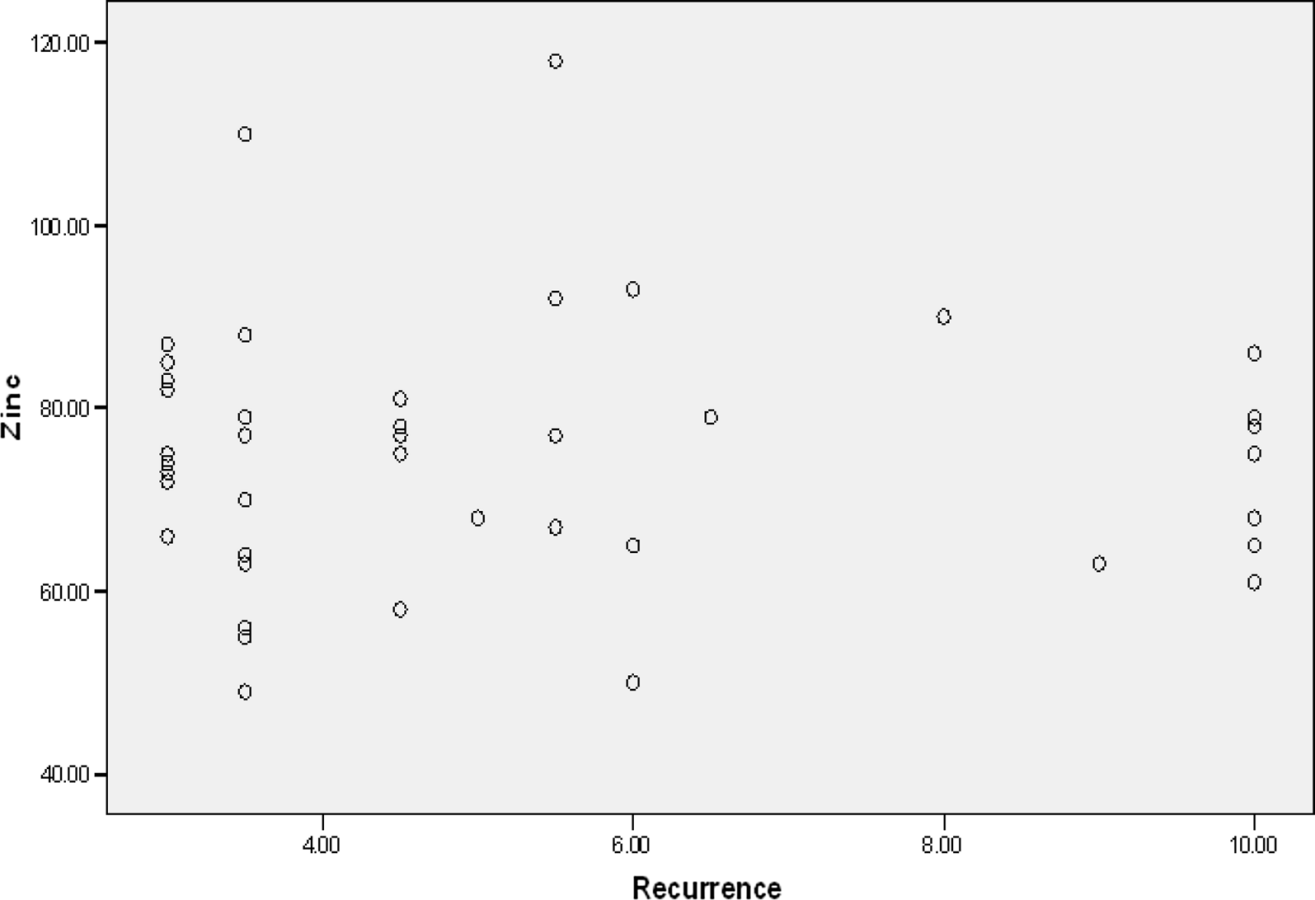
Correlation of serum zinc level (μg/dL) in comparison with rate of recurrence per year in herpes cases

Moreover, zinc level was not significantly different regarding the patients' age, gender, and the number of recurrences in a year (*p* = 0.953, *p* = 0.336, *p* = 0.123). However, a significant relationship was observed between the amount of zinc and the duration of the last recovery period. Individuals with a higher serum zinc level (*p* = 0.009), experienced a shorter duration of lesions (Table 4) (Figs. 1 and 2).

**Table 4:** The relationship among serum zinc level, duration, and recurrence of lesions

Herpes (N = 43)	Minimum	Maximum	Mean	SD	Pearson correlation	*p* value*
Duration of lesions	7.5 days	15.5 days	10.0581 days	2.13589	− 0.391	0.009
Recurrence rate	3 times a year	10 times a year	5.2674	2.51270	− 0.009	0.953

^*^p value < 0.05 is considered as significant

The ES for comparing serum zinc level between cases and controls and two genders were small (ES = 0.009, ES = 0.297). The ES values were small for all comparisons, except for duration of lesions (r = 0.391). The power value based on our sample size was highly acceptable (96%), that shows a sufficient sample size for comparing serum zinc levels in RHL individuals and healthy subjects.

## Discussion

The results of the present study show that, serum zinc level was not significantly different in the RHL patients compared to healthy individuals. Furthermore, age, gender, and the rate of recurrence had no significant relationships with zinc level. The interesting point is that, the duration of lesions was significantly longer in the patients with low serum zinc levels.

The reason that, this element may have an effect on reducing the recovery time of this lesion may be recognized as its various effects on the human body. Zinc is an essential element for the natural function of cells, tissues, and organs of the body. As a factor, it results in the enhancement and migration of keratinocytes during the process wound healing [12, 13] In regard to wound healing, it is found that Zinc has a major role in regulating every phase of this process. It is involved in membrane repair, oxidative stress, coagulation, inflammation and immune defense, tissue re-epithelialization, angiogenesis and fibrosis/scar formation [14]. Additionally, in relation to host immunity, Zinc has the capacity to safely control the viruses by stimulating cellular immunity. T-Lymphocyte response is a cellular immune based immunity (CMI), which is considered to be important in protecting against viral, fungal, and protozoic infections, as well as against malignant and autoimmune diseases. Zinc appears to increase the number of the effector and helper T-cells, either the precursors of the antibody-forming cells or the increased activity of suppressive cells [13, 18, 24]. In a study conducted by Barman et al. [25], Zinc deficiency has been found to reduce host immunity by decreasing circulating T cells and the phagocyte activity of other cells, which can generally affect cell-mediated immunity.

According to the above, In herpes labialis infection in addition to the need for virus eradication the wound healing process is highly active [3]. Therefore, it seems that both functions of zinc element mentioned are greatly involved in curing this lesion.

Although zinc deficiency may have many effects, clinical evaluation of this deficiency is not easy, because its signs and symptoms are non-specific. Those signs associated with zinc deficiency are as follows: decreased plasma zinc levels (< 70 μg / dL), retinal alcohol in the retina (leading to night blindness), T-lymphocyte dysfunction, decreased collagen synthesis (resulting in poor wound healing), and decreased RNA polymerase activity in different tissues [12, 23].

Recently, the role of zinc compounds has been considered in the prevention and control of many diseases and abnormalities related to humans and animals. The effectiveness of zinc compounds on making the local defense system stronger, reducing the inflammation of the bacteria, and producing epithelial cells and endothelium vessels in the repair of foot injuries has been proven. Many studies indicated that, administration of systemic or topical zinc helps in reducing the recovery rate of RHL [15–17, 20, 26].

In a study conducted by Khozeimeh et al. [22], salivary zinc was compared between the 40 people with RHL under both conditions of disease and recovery (21 days after the improvement of lesions of Herpes) and 40 healthy people. The result showed that, zinc level was significantly different in disease state compared to recovery stage, and also in recovery compared to healthy subjects. In this study, there was no significant correlation among zinc levels and the patients' age, gender, recurrence rate, and duration of healing. In regard to sample size and inclusion criteria this study was similar to our study. However, in our study, serum zinc level was not significantly different in the subjects with a history of herpetic lesions compared to healthy individuals. One of the reasons for this difference is that, saliva is probably not an exact criterion for measuring the amount of zinc in the body. Also, a study conducted by Freeland-Graves et al. in 1981, compared whole saliva and salivary sediment after the administration of a low zinc diet (3.2 mg/day) in 12 women for 22 days. The zinc level of saliva and salivary sediment (centrifuged saliva) were measured before and after the start of the study. The results showed that, zinc saliva levels remained constant during the study, while zinc levels significantly increased in salivary sediment by passing 22 days from the administration. As a result, complete saliva can be considered by no means as an acceptable benchmark for the evaluation of zinc intake [27]. It is noteworthy that, in several studies, it has been determined that serum zinc is the most widely used biomarker for assessing the status of zinc in the population. Serum zinc concentration indicates recent consumption of zinc or regular zinc intake, indicating that, in populations with low zinc diet, the serum zinc level is lower, which shows a high zinc deficiency risk. These studies showed that, the best time to measure serum zinc is whilst fasting in morning [23, 28].

In a study conducted by Brody et al. [29], a zinc sulfate solution was used on the site of herpes lesions on the skin and the oral mucosa (used on the skin at a concentration of 0.05–0.025% and on the mucus at a concentration of 0.01–0.025%). This study also showed that, the treatment with this solution on the skin prevents post-herpetic erythema multiform in addition to its herpes preventive role.

In a laboratory study conducted by Max Arens et al. [30], the effects of different concentrations of different zinc salts (zinc acetate, zinc lactate, zinc sulfate, zinc gluconate) on isolated HSV virus in culture medium were investigated. The results showed that, the isolated HSV in laboratory conditions, is effectively inactivated by the treatment with Zinc salts and the degree of inactivation of the virus depends on the type of HSV, zinc concentration, and treatment length.

In another article published by Femiano et al. [31], they examined the effect of zinc on RHL. In this paper, 20 patients (including 12 women and 8 men) with an average age of 26.6 years old with a history of RHL more than 6 times per year and a recovery period of more than 8–14 days, were treated by zinc sulfate 22.5 mg twice daily for 4 months. The patients were followed-up for 12 months. The study concluded that, herpes ulcers decreased by less than 4 times a year and the recovery period reduced to less than 7 days at each relapse. As a result, this study showed that, systemic zinc sulfate reduced the relapse rate and the period of RHL. However, our study did not address the treatment of such individuals.

In a study published by Antoine et al. [32], ZOTEN (zinc oxide tetrapod nanoparticle) was used as an intravaginal vaccine in female rats. The ability to inhibit HSV-2 by ZOTEN reduced the clinical symptoms and lowered the mortality rate in rats. ZOTEN inhibited recurrent infection by increasing T cell-mediated and antibody-mediated response. Overall, the effect of the vaginal microbial vaccine on primary and secondary vaginal herpes virus infection was determined.

Individuals suffering from other mucosal disease such as oral lichen planus, recurrent aphthous stomatitis, xerostomia and burning mouth syndrome have also been recognized as having lower serum zin concentrations compared to healthy individuals [28, 33]. In the case of other viral diseases and body minerals, in a study conducted by Okwara et al. [34], 51 adult patients with human immunodeficiency viral infection (HIV) and 48 healthy people were included. Selenium, zinc, and magnesium were also measured. All the minerals were lower among the HIV-infected individuals compared to healthy subjects. Also, Raza et al. [35] showed that zinc deficiency is related to the recurrence, persistence and progression of human papilloma virus lesions known as viral warts.

Noteworthy, due to the fact that, herpes is a common and self-limiting disease, and referral to a dermatologist or dentist is usually rare; therefore, patient selection was somehow difficult. Also, encouraging the patients for blood sampling was not easy. So, further studies with larger sample sizes are suggested. It is also suggested that, in future studies, laboratory confirmation of HSV1 and HSV2 should be considered prior to serum zinc level evaluation to detect the positive asymptomatic patients. Moreover, other viral diseases should be checked regarding the zinc level. It is reasonable to simultaneously compare the saliva and serum levels. Comparison of serum zinc levels in both disease and recovery state can also be beneficial.

## Conclusion

The results of this study indicate that, zinc deficiency can be a risk factor for increasing the duration of recovery period of herpes labialis. Therefore, the evaluation of serum zinc level in the individuals with a history of recurrent herpes labialis and subsequent administration of zinc might be beneficial. Further studies are warranted to profoundly evaluate this issue.

## Data Availability

The data supporting the findings of this study are available upon reasonable request from the corresponding author. However, restrictions were applied to the public availability of these data, because of the patient's confidentiality.

## Abbreviations

RHL: Recurrent herpes labialis
HSV: Herpes simplex virus
ZnO: Zinc oxide
ES: Effect size
CMI: Cellular immune based immunity
ZOTEN: Zinc oxide tetrapod nanoparticle
HIV: Human immunodeficiency virus
